# Aspirin versus LMWH for VTE prophylaxis after orthopedic surgery

**DOI:** 10.1515/med-2023-0760

**Published:** 2023-08-26

**Authors:** Qingqing Wei, Jing Sun, Yusuo Bai, Chang Meng, Guobin Miao, Peng Liu, Haijun Wang

**Affiliations:** Department of Critical Care Medicine, Emergency General Hospital, Beijing, 100028, PR China; Department of Vascular Surgery, Chuiyangliu Hospital Affiliated to Tsinghua University, Beijing, 100022, PR China; Department of Emergency, Emergency General Hospital, Beijing, 100028, PR China; Department of Cardiology, Ordos Central Hospital, Ordos School of Clinical Medicine, Inner Mongolia Medical University, 23 Yijinhuoluo West Street, Dongsheng District, Inner Mongolia, 017000, PR China; Department of Emergency, Emergency General Hospital, XiBaHe South Road 29, Chaoyang District, Beijing, 100028, PR China

**Keywords:** aspirin, low-molecular-weight heparin, venous thromboembolism, joint surgery

## Abstract

Low molecular weight heparin (LMWH) is often used to prevent perioperative venous thrombosis after surgery, but aspirin is also recommended by academics. Studies were searched in electronic databases until February 24, 2023. We performed a meta-analysis to evaluate the safety and efficacy of aspirin and LMWH for venous thromboembolism (VTE) prophylaxis in patients after orthopedic surgery. The outcomes were death from any causes, deep vein thrombosis (DVT), pulmonary embolism (PE), etc. This study was registered with INPLASY, number 202320117. Six randomized controlled trials enrolled 13,851 patients with postoperative joint surgery. The risk of DVT was comparable between the two groups when aspirin was combined with mechanical devices (RR 0.61 [95% CI 0.27–1.39], *I*² = 62%, *P* = 0.24). No significant differences in all cause death, PE, wound infection, and wound complication were found between the aspirin and LMWH groups. In this meta-analysis, the mortality rate was comparable between the aspirin and LMWH groups. However, aspirin alone had a higher risk of DVT than LMWH. Based on the results of this meta-analysis, we suggest aspirin combined with mechanical devices for VTE prophylaxis in patients after orthopedic surgery.

## 1 Introduction

Venous thromboembolism (VTE) is a serious consequence in patients with orthopedic trauma [1]. Some clinical guidelines recommend the use of thromboprophylaxis after orthopedic surgery to reduce the risk of VTE after orthopedic surgery and to reduce the associated risk of death and complications [2,3].

Previous studies have analyzed the efficacy and safety of aspirin and low molecular weight heparin (LMWH) in different orthopedic patients [4]. Recent large randomized clinical trials (RCTs) [5] have filled a gap in antithrombotic therapy in patients with surgically treated fractures. The results of most studies indicate that aspirin and low molecular weight have similar outcome markers, but the sample sizes of most studies are relatively small. Some studies have also analyzed the advantages of aspirin combined with mechanical devices to prevent venous thrombosis. After all, aspirin as an oral drug has irreplaceable convenience compared with LMWH, but LMWH as a clotting pathway inhibitor also plays a very important role in thrombosis.

O’Toole et al. [5] included patients who had had surgery for a broken limb or had any pelvic or acetabular fractures at multiple centers, and the results showed that aspirin’s thromboprophylaxis was no less effective at preventing death than LMWH. Anderson et al. [6] included total hip arthroplasty patients, and by extending the application time of aspirin to 28 days, compared with the LMWH group, the incidence of deep vein thrombosis (DVT) was similar between the two groups. The author mentioned that considering economic factors, aspirin could also be considered for clinical application.

Several subsequent studies [7–10] have looked at similar issues in patients undergoing joint replacement surgery, but with different drug timings and follow-up times. Recent studies [11] have analyzed the effect of other anticoagulant drugs on the prevention of DVT, which is also the direction of future research. Three studies [8–10] combined with mechanical devices, also offer new solutions for future treatment. In this meta-analysis, we summarized previously published RCTs to investigate the efficacy and safety of aspirin and LMWH in antithrombotic therapy for patients after orthopedic surgery.

## 2 Methods

We carried out the meta-analysis in accordance with the Preferred Reporting Items for Systematic Reviews and Meta-analyses guidelines [12]. Our protocol has been registered on the International Platform of Registered Systematic Review and Meta-analysis Protocols database (Inplasy protocol: INPLASY202320117), and is available in full on inplasy.com (https://inplasy.com/inplasy-2023-2-0117). Ethics approval was not required for our work.

### 2.1 Search strategy

Three independent researchers conducted extensive electronic searches for relevant articles published until February 24, 2023. The database includes PubMed, Embase, and the Cochrane database. Manually select relevant randomized controlled trial. The search strategy of the literature is shown in Table A1.

### 2.2 Inclusion and exclusion

EndNote (X9 version) software is selected for document management; two investigators independently evaluated the eligibility of the identified items. The title and summary are filtered for the first time, and qualified articles are reserved for full-text review. Inclusion criteria for studies meeting the following requirements include: (1) patients after postoperative joint surgery, (2) treat with aspirin or LMWH, and (3) outcome indicators: all cause death, DVT, pulmonary embolism (PE), wound infection, wound complication, including one. We excluded studies enrolling patients <18 years old, and there was not enough data to extract, such as the summary of some meetings, literature materials such as review and pharmacological introduction. We contacted the authors if associated data from their studies were required.

### 2.3 Bias and quality assessment

The two researchers independently evaluated, preliminarily selected and checked the literature data according to the unified and standardized method, and included them in the literature in strict accordance with the admission and exclusion criteria, and then collected information. Evaluate the quality of selected articles according to the quality evaluation standard of Cochrane Reviewer Handbook 5.1.0 [13].

### 2.4 Data synthesis and analysis

Revman5.3 was used for meta-analysis. Data which met homogeneity (*P* > 0.10 and *I* ^2^ ≤ 50%) through heterogeneity test were meta-analyzed using fixed effect model. If homogeneity (*P* ≤ 0.10 or *I* ^2^ > 50%) was not met, and heterogeneity cannot be ruled out, random effect model can be used to combine effects [14]. While it should be noted that sensitivity analysis and subgroup analysis should be considered for this type of analysis data. For the continuous outcomes, mean differences and 95% CIs were estimated as effective. Some included RCTs reported median as the measure of treatment effect, with interquartile range. A *P* value <0.05 was considered statistically significant.

## 3 Results

**Figure 1:**
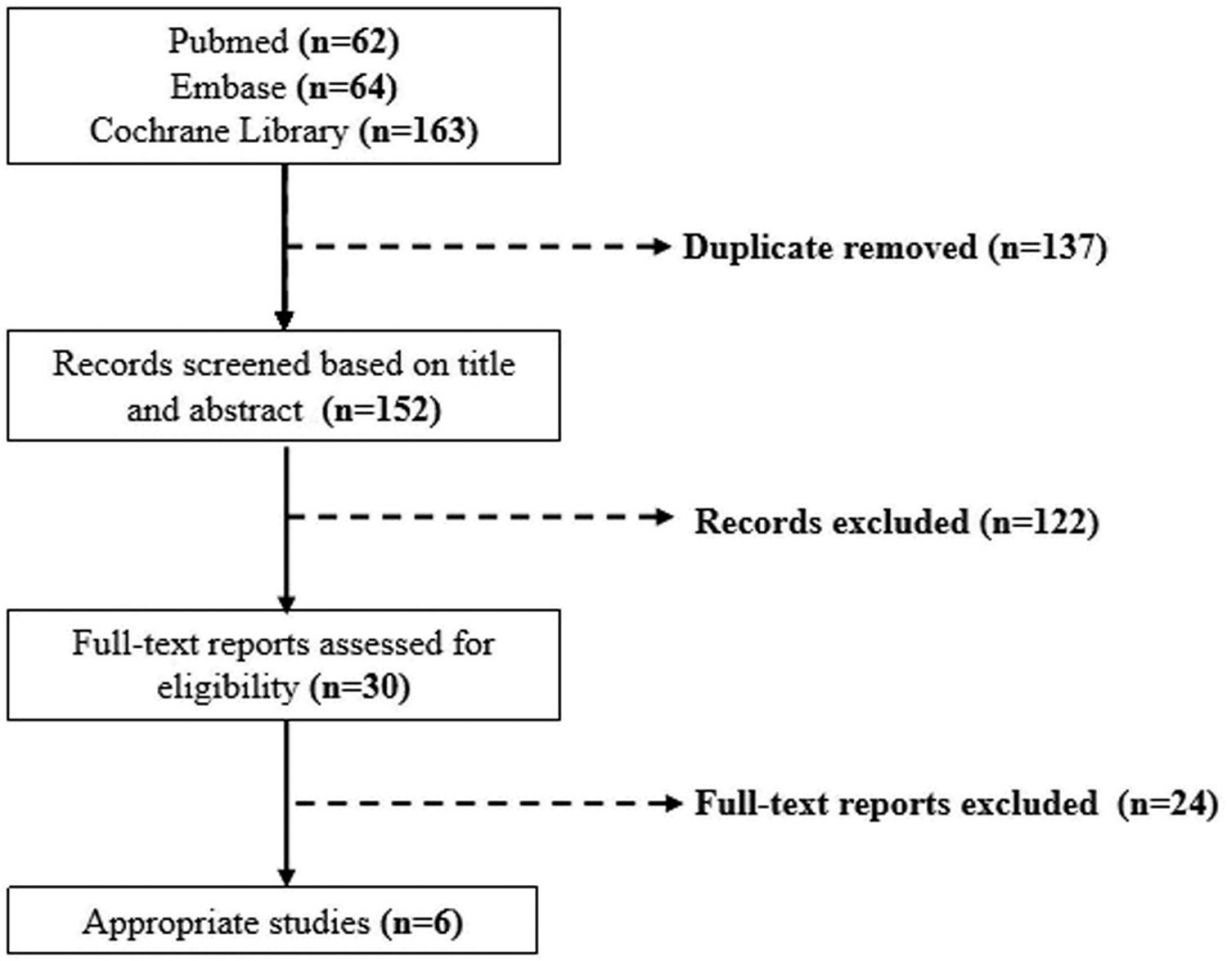
The flow chart of the search and study selection process.

The flow chart (Figure 1) summarizes the search and study selection process. A total of 289 studies were identified through the electronic searches, of which 137 were excluded due to duplication. Around 122 studies were also excluded after reading the titles and abstracts. The remaining 24 studies were assessed by reading the full texts. Data from six trails of 13,851 patients evaluating the efficacy and safety in postoperative joint surgery treated with aspirin versus LMWH were included.

The main features of the included trials are presented in Table 1. All included studies were randomized controlled trials, and the follow-up time lasted from hospitalization to 6-week or 3-month. Three of the six trials (*n* = 633) included patients treated with aspirin combined mobile compression device, subgroup analysis of DVT, and wound complications were performed. No differences were observed in terms of the proportion of patients lost to follow up between the aspirin and LMWH groups across trials.

**Table 1:** Design and outcomes of the studies included in the meta-analysis

Num.	Author/Year	Design	Intervention assignments		Participants				Outcomes
			Aspirin	LMWH	Sample size, *n*	Mean age, years (A/L)	Male% (A/L)	Time of medication	
1	Robert/2023	RCTs, MC	81 mg bid	Enoxaparin 30 mg twice daily	12,211	44.5/44.7	62.8/61.7	About 1 month	All cause deaths, PE, DVT, bleeding complication, wound complication, infection (follow 90-day)
2	Zou/2014	RCTs, SC	100 mg qd	AxaIU 4,000 U qd	222	62.7/65.7	25.5/17.9	14 days	DVT, wound complications, limb swelling (follow 4-week)
3	Anderson/2013	RCTs, MC	81 mg qd	Dalteparin 5,000 U qd	785	57.6/57.9	60/53.3	28 days/10 days	All cause deaths, PE, DVT, major bleeding, minor bleeding, wound infection (follow 90-day)
4*	Jiang/2014	RCTs, SC	100 mg qd	5,000 U qd	120	65.1/63.8	8.3/6.7	14 days	All cause deaths, DVT, ematoma, wound complications (follow 6-week)
5*	Colwell/2010	RCTs, MC	81 mg qd	30 mg Bid until discharge and then 40 mg qd	392	63/62	45/46	10 days	DVT; hematoma (follow 12-week)
6*	Gelfer/2006	RCTs, SC	100 mg qd	Enoxaparin 40 mg qd	121	68/67	34/38	In-hospital	All cause deaths, PE, DVT, wound drainage (follow 3-month)

A/L = aspirin group/LMWH group; Bid = twice daily; DVT = deep vein thrombosis; LMWH = low molecular weight heparin; MC = multicenter; PE = pulmonary embolism; PS = prospective study; qd = Once a day; RCTs = randomized clinical trials; SC = single center; *Aspirin combined mobile compression device.

**Figure 2:**
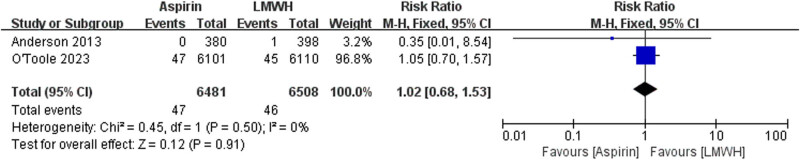
The outcomes of all cause death.

**Figure 3:**
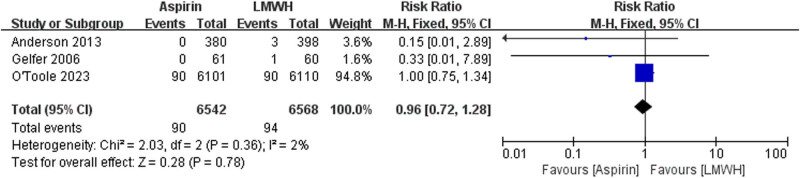
The outcomes of PE.

**Figure 4:**
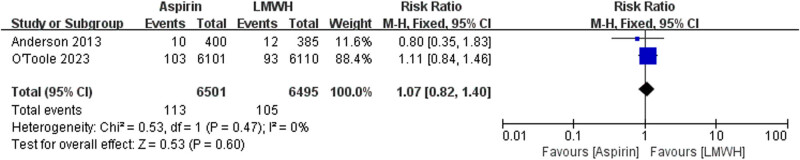
The outcomes of wound infection.

The data of all cause death were available from two trials (Figure 2). There is no significant differences between the aspirin and LMWH groups (RR 1.02 [95% CI 0.68–1.53], *I*² = 0%, *P* = 0.91). The data of PE were available from three trials (Figure 3). No significant differences were found between the aspirin and LMWH groups (RR 0.96 [95% CI 0.72–1.28], *I*² = 2%, *P* = 0.78). The data of wound infection were available from two trials (Figure 4). No significant differences were found between the aspirin and LMWH groups (RR 1.07 [95% CI 0.82–1.40], *I*² = 0%, *P* = 0.60).

**Figure 5:**
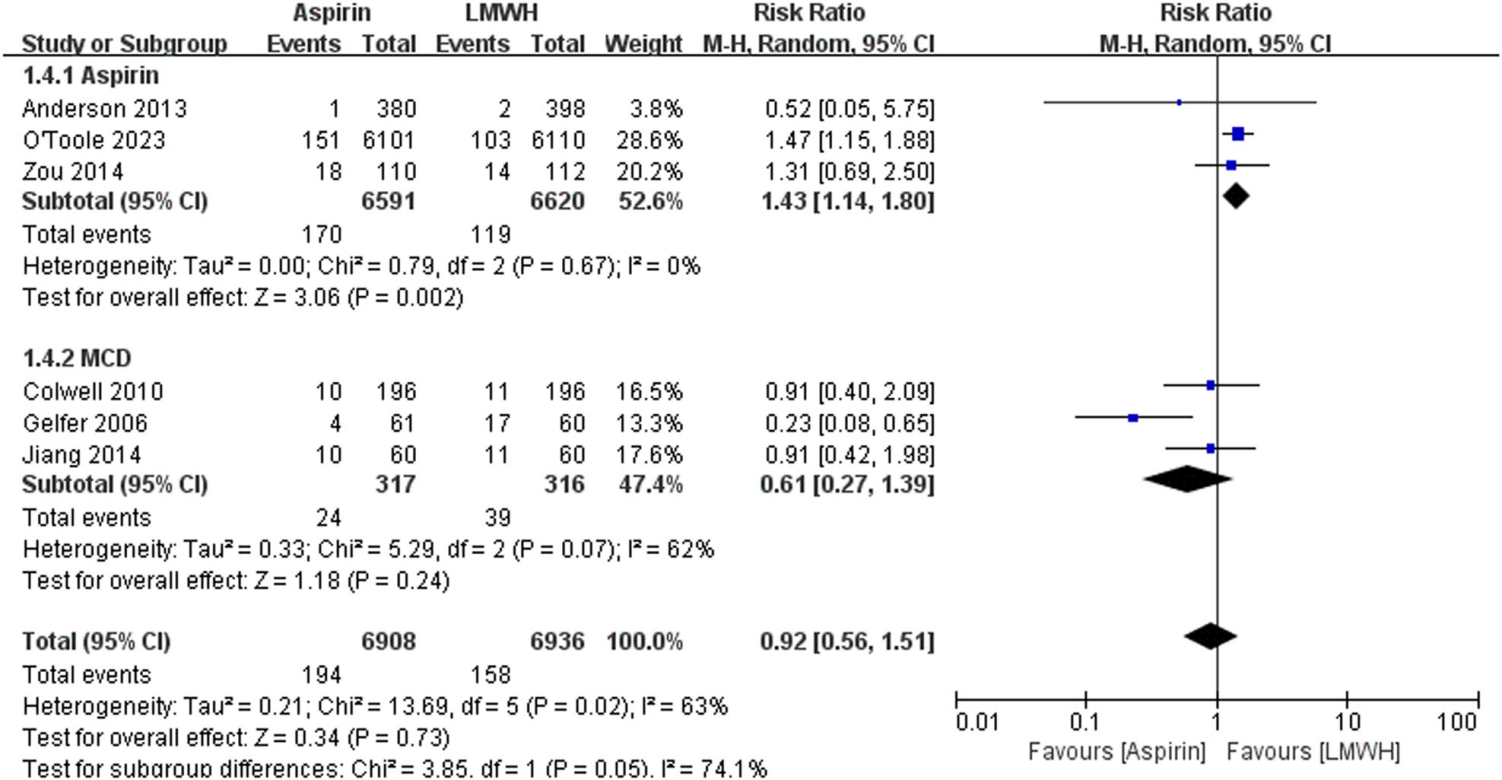
The outcomes of DVT.

**Figure 6:**
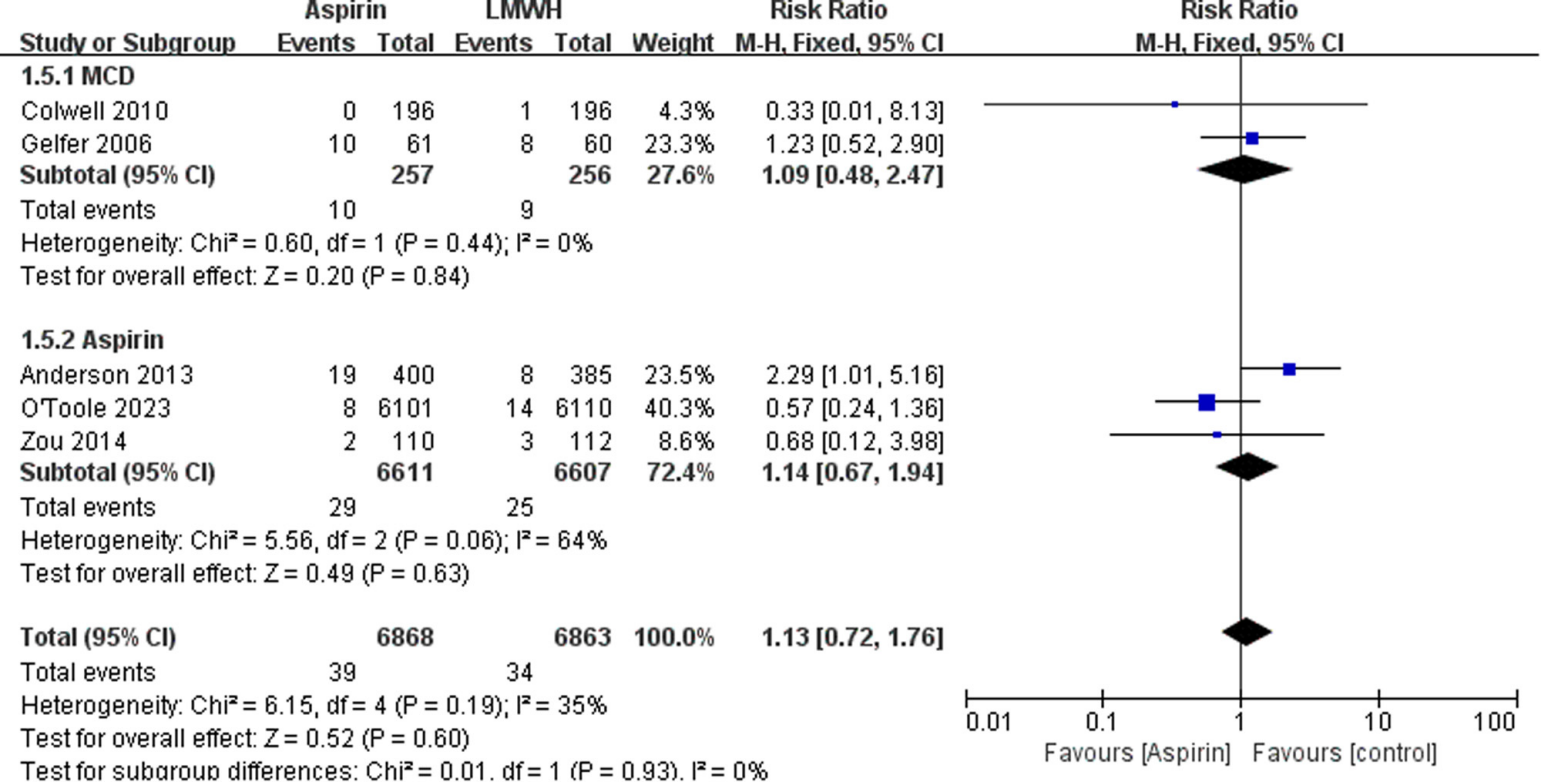
The outcomes of wound complication.

The data of DVT were available from six trials (Figure 5). There is no significant differences between the aspirin and LMWH groups (RR 0.92 [95% CI 0.56–1.51], *I*² = 63%, *P* = 0.73). In the subgroup analysis, we saw that the risk of DVT in the aspirin alone group was higher than that in the low molecular heparin group (RR 1.43 [95% CI 1.14–1.80], *I*² = 0%, *P* = 0.002), but the risk of DVT was comparable between the two groups when aspirin was combined with mechanical devices (RR 0.61 [95% CI 0.27–1.39], *I*² = 62%, *P* = 0.24). The data of wound complication were available from five trials (Figure 6). There is no significant differences between the aspirin and LMWH groups (RR 1.13 [95% CI 0.72–1.76], *I*² = 35%, *P* = 0.60). There was no statistically significant difference between aspirin alone and aspirin combined with mechanical devices (RR 1.14 [95% CI 0.67–1.94], *I*² = 64%, *P* = 0.63; OR 1.09 [95% CI 0.48–2.47], *I*² = 0%, *P* = 0.84).

We used Revman to investigate the influence of a single study on the overall pooled estimate of each predefined outcome. We found that the removal of any one study would not affect the following results. The results of the risk of bias assessment with the RoB2 of randomized control trials are summarized in the Table A1.

## 4 Discussion

Studies have reported that approximately 1.5 million hip and knee arthroplasty procedures are performed each year in the United States [15,16]. The incidence of surgical symptomatic VTE in patients is about 2%, posing a serious threat to postoperative recovery [11,17]. The prevention of DVT has become the focus of many scholars. LMWH has traditionally been used for anticoagulant therapy. In recent years, the use of aspirin-based thromboprophylaxis has increased [18,19]. This meta-analysis discussed the efficacy and safety of aspirin and LMWH in patients after orthopedic surgery by summarizing several RCTs. Our article had a large sample size and high quality, and the results were very reliable.

However, regarding aspirin in the prevention of DVT in patients after orthopedic surgery, the efficacy of aspirin as the only prophylactic drug is also questioned [20], which is consistent with the results of this study. In this meta-analysis, we could see that there was no statistical difference between the aspirin group and the LMWH group in the prevalence of PE and wound complications. In terms of the incidence of DVT, we can see that aspirin alone is worse than LMWH, and aspirin combined with mechanical device changes this outcome. This may seem different from the conclusions of individual studies, but it is probably the most realistic conclusion because our analysis included a large sample size and relied on standardized statistical analysis, after all, aspirin is more commonly used as an antiplatelet agent for the prevention of arterial embolization events. Therefore, we suggest that aspirin combined with mechanical auxiliary devices can be considered in the prevention of DVT after orthopedic surgery. On the one hand, aspirin can reduce the pain of patients, and it is easy to operate and implement. Future research can further explore the advantages and disadvantages of different mechanical devices, and provide a more simple and feasible program for patients after fracture surgery. Recent studies compared the risk of thromboembolic events under different routes of administration, which also provides some inspiration for this study [21]. There are also studies that female patients have a low risk of gynecological surgery embolization events, and gender classification is also one of the future research directions [22]. The metabolic pathways of embolic events have also been studied, which may also be one of the future research directions [23].

There are several limitations to be mentioned. First, we included several RCTs, in which different populations had different oral aspirin doses, medication cycles, and follow-up times, which may have increased the heterogeneity of the findings. Second, the number of patients was relatively small and some follow-up outcomes could not be obtained. For example, the cerebral function in different oxygen supply strategies could not be evaluated for cardiac arrest patients.

## 5 Conclusion

Death rates were comparable between the aspirin group and the LMWH group. Based on the results of this meta-analysis, we recommend the use of aspirin in combination with mechanical devices for the prevention of VTE in patients after orthopedic surgery.

## Figures and Tables

**Figure A1:**
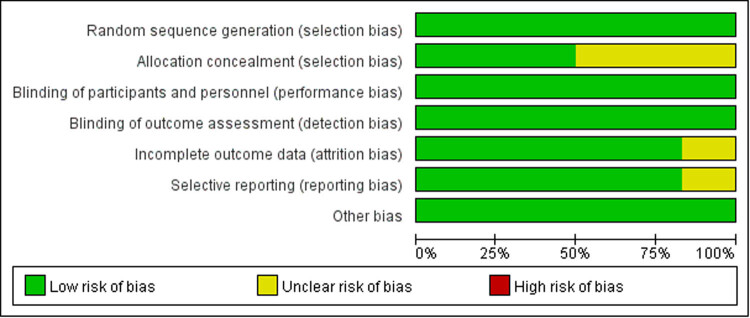
Risk of bias graph.

## Appendix

**Table A1:** Search strategy

Electronic database	Search strategy
PubMed (NCBI)	(“aspirin”[Title/Abstract] OR “acetylsalicylic acid”[Title/Abstract] AND (“Low molecular weight heparin”[Title/Abstract] OR “lmwh”[Title/Abstract] OR “nadroparin”[Title/Abstract] OR “Lovenox”[Title/Abstract] OR “heparin”[Title/Abstract]) AND (“cataclasis”[Title/Abstract] OR “fracture”[Title/Abstract] OR “joint”[Title/Abstract] OR “articulation”[Title/Abstract] OR “arthroplasty”[Title/Abstract]) AND (“randomized controlled trial” OR “randomized” OR “placebo” OR “randomly” OR “trial”)
Embase	#1 (‘aspirin’:ab,ti OR ‘acetylsalicylic acid’:ab,ti)
	#2 (‘Low molecular weight heparin’:ab,ti OR ‘lmwh’:ab,ti OR ‘nadroparin’:ab,ti OR ‘Lovenox’:ab,ti OR ‘heparin’:ab,ti)
	#3 (‘cataclasis’:ab,ti OR ‘fracture’:ab,ti OR ‘joint’:ab,ti OR ‘articulation’:ab,ti OR ‘arthroplasty’:ab,ti)
	#4 (‘randomized controlled trial’:ab,ti OR ‘randomized’:ab,ti OR ‘placebo’:ab,ti OR ‘randomly’:ab,ti OR ‘trial’:ab,ti)
	#5 #1 AND #2 AND #3 AND #4
Cochrane	(aspirin OR acetylsalicylic acid) AND (lmwh OR Low molecular weight heparin OR nadroparin OR Lovenox OR heparin) AND (cataclasis OR fracture OR joint OR articulation OR arthroplasty)
